# Physician’s autonomy in the face of AI support: walking the ethical tightrope

**DOI:** 10.3389/fmed.2024.1324963

**Published:** 2024-03-28

**Authors:** Florian Funer, Urban Wiesing

**Affiliations:** Institute for Ethics and History of Medicine, University Hospital and Faculty of Medicine, University of Tübingen, Tübingen, Germany

**Keywords:** ethics, autonomy, (shared) decision-making, professional responsibility, clinical decision support systems, machine learning, artificial intelligence

## Abstract

The introduction of AI support tools raises questions about the normative orientation of medical practice and the need to rethink its basic concepts. One of these concepts that is central to the discussion is the physician’s autonomy and its appropriateness in the face of high-powered AI applications. In this essay, a differentiation of the physician’s autonomy is made on the basis of a conceptual analysis. It is argued that the physician’s decision-making autonomy is a purposeful autonomy. The physician’s decision-making autonomy is fundamentally anchored in the medical ethos for the purpose to promote the patient’s health and well-being and to prevent him or her from harm. It follows from this purposefulness that the physician’s autonomy is not to be protected for its own sake, but only insofar as it serves this end better than alternative means. We argue that today, given existing limitations of AI support tools, physicians still need physician’s decision-making autonomy. For the possibility of physicians to exercise decision-making autonomy in the face of AI support, we elaborate three conditions: (1) sufficient information about AI support and its statements, (2) sufficient competencies to integrate AI statements into clinical decision-making, and (3) a context of voluntariness that allows, in justified cases, deviations from AI support. If the physician should fulfill his or her moral obligation to promote the health and well-being of the patient, then the use of AI should be designed in such a way that it promotes or at least maintains the physician’s decision-making autonomy.

## 1 Introduction

Artificial intelligence and considerations about its suitability for different clinical contexts are on everyone’s lips in the medical field. The term artificial intelligence (AI) usually refers to forms of machine learning, that is, algorithms whose mode of operation is based on extracting patterns and correlations from large amounts of data, synthesizing models from them and making accurate probability statements for new input data on this basis. At first glance, this mode of operation seems to be perfectly suited to the manifold need for predictive statements in diagnostics, prognostics, and therapeutics. Based on very large data sets, the consideration of which exceeds the capabilities of individual healthcare professionals by far, predictive statements could become more accurate. The potential of integrating AI into health care as a positive contribution to patient care therefore seems very promising (1). Hence, AI is being developed primarily in form of clinical decision support systems (CDSS) (2), which match AI models trained on big data with individual cases at hand and give the clinician specific information about what is statistically most likely to be found or most likely to be done.

For the field of medicine, the question arises of who should retain control for clinical decisions in general and for such automated decisions in particular. From a normative point of view, the answer to this question has far-reaching implications, for example for the requirements for reevaluation of automated recommendations, the bearing of accountability and liability for their use and any harm resulting from them, as well as the necessary demands on information and communication. If one looks beyond the horizon into other areas of society, one will find parallels to discourses on the use of autonomous weapons (3) or autonomous driving (4). It is therefore not surprising that attempts for solutions in other areas are also sometimes discussed for medical practice (3, 5). Unlike in other areas, however, medical practice has to be oriented toward the individual health and well-being, thereby respecting the preferences of patients due to the high value of individual self-determination. In order to make this possible, there are special requirements for the use of AI in healthcare, which particularly concern the clinician’s scope of action and his or her decision-making autonomy—with the help of or despite the use of AI support. Preliminary empirical results of the past years, especially from qualitative interview studies with doctors, underline the relevance of the topic of physician’s autonomy and related concepts like responsibility, control, and decision-making authority (6–13). According to the arguments put forward by interviewees (7, 8, 10) as well as within theory-building (2, 14), there are potential risks to the physician’s autonomy such as “de-skilling,” i.e., the gradual loss through non-use or reduced relevance of the use of certain skills, or the inability to provide adequate information about risks and possible errors, for example due to biases. The interdisciplinary discussion of recent years has led to the widely shared consensus that AI should only be used, at least for health care of individuals, in such a way that it supports human decision-making, but in no way replaces it (15). For example, in the area of image-based diagnostics there are various proposal for AI tools, which could help for the radiological assessment of CT images or ophthalmoscopies by suggesting potentially relevant abnormalities to the physician, so that he or she can verify it. Other proposals for AI tools aim to provide intraoperative navigation for surgeons or to support physicians with the selection of suitable therapeutic approaches for chronic diseases. But most of these proposals have common that the decision-maker must remain a human being, for our context: the physician in charge together with the patient being treated. In order to achieve this, we argue, the autonomy of the physician is required.

The autonomy of the physician has several meanings today. First, it must be distinguished from the autonomy of the medical profession. This medical profession has the freedom to regulate certain professional matters itself and to set professional standards [c.f. on this topic (16)].^1^ However, this is to be distinguished from the individual decision-making autonomy of the physician, which is the issue here. This means, the physician’s freedom to make a judgment about what is medically indicated in the given situation and to propose it to the patient. For the purposes of this essay, it is always assumed that the medical decision follows a process of shared decision-making with the patient. Next to it: There is nothing to suggest that medicine should change its normative orientation because of AI support. There is a strong case to be made that there will be problems in applying AI to comply with existing norms and values. However, this does not mean that the normative orientation is therefore insignificant or in need of change. Rather, the use of AI must take place in such a way that it is consistent with the existing norms and values.

Using AI support as an example, we discuss the relevance of physicians’ individual autonomy to the process of clinical decision-making. For this purpose, we conducted a conceptual analysis of physicians’ autonomy discussing it with the relevant literature on AI in healthcare. The aim of our analysis was to identify such aspects that point to the necessity and/or relevance of the physician’s decision-making autonomy for the use of AI in healthcare. Based on these aspects, ethical implications of the gain or loss of clinicians’ autonomy for clinical decision-making were discussed. The sections of this paper each argue for the following hypotheses:

1. The physician’s (professional) decision-making autonomy is an expedient means to fulfill the moral obligation to promote the health and well-being of the individual patient, with utmost respect for the patient’s autonomy and rights.
2. The use of AI support may compromise the way medical (professional) decision-making autonomy is exercised within clinical decision-making, …
  (a) due to the inherent way AI works and the physician’s challenge of assessing its statements in terms of their adequacy and action-guiding justification;
  (b) due to the physician’s challenge to integrate AI support statements into shared decision-making with the patient; and
  (c) due to the implementation in structural and institutional contexts and the physician’s challenge to decide for/against the AI support’s use and its statements.
3. If the physician should fulfill his or her moral obligation to promote the health and well-being of the patient, with the utmost respect for the patient’s autonomy and rights, then the use of AI should be designed in such a way that it promotes or at least maintains the physician’s (professional) decision-making autonomy.

## 2 The decision-making autonomy of physicians: the wherefores and potential misunderstandings

The physician’s autonomy in decision-making finds its starting point in the physician-patient relationship (17, 18). As with any other ethical relationship, the relationship between physicians and patients is accompanied by mutual moral obligations on the part of the persons involved (18). Physicians have knowledge and skills that give them the power to help people in vulnerable life situations (17). But with great power comes great responsibility in the form of moral (and sometimes legal) obligations. It is the moral obligation of the physician to arrange both the conditions of the practice of medicine and the services provided in such a way that the main ends of medical practice, the promotion of the health and well-being of the patient, is applied as rationally, efficiently and safely as possible in accordance with the physician’s knowledge and skills. This requires the decision-making autonomy of the physician.

The physician’s individual decision-making autonomy must be differentiated into personal and professional decision-making autonomy (18).

The physician cannot be reduced to his or her professional membership alone, but also has the right as a human being to be respected in his or her autonomy. Ensuring physician’s personal decision-making autonomy does justice to his or her capacity as person to make personal choices and to follow his or her knowledge and conscience about what is considered by him or her as morally acceptable and what constitutes ‘good medicine’ (18). The physician cannot therefore be expected to act contrary to his or her own personal beliefs, values, or commitments. Specifically, this relates primarily to cases of deep moral dilemmas and divergent beliefs in medicine, in which the physician may refuse a legally permitted intervention on grounds of conscience “if the individual patient is not harmed or discriminated against and if the patient’s health is not endangered” [International Code of Medical Ethics (ICoME), No.29] (19). The typical examples of conscientious objection are abortion and euthanasia. The physician is therefore (with the exception of urgent emergency situations) free, in deference to his or her personal decision-making autonomy, to step out of the professional relationship and, for example, refer the seeming patient to other physicians. This aspect of the autonomy of a physician will not be influenced by AI.

However, the actual interest of this article is the physician’s professional decision-making autonomy insofar as it is part of the professional physician-patient relationship. But why should physicians be allowed any autonomy at all in decision-making with their patients? In order to answer this question, one can refer to the most basic principles of medical ethics. Since its origins, medical practice has been inscribed with a teleology, that is, the orientation toward ethical goals to be followed. According to today’s teleology, it can be said that the goal of medical practice is to promote the patient’s health and well-being, with utmost respect for human dignity and, consequently, the patient’s autonomy and rights.^2^ Satisfying this goal requires the physician morally to orient his actions as closely as possible to the patient’s individual case with its circumstances. The decision-making autonomy of physicians and the freedom to care for their patients without interference (except in training situations) can be understood in this sense as an expedient means to appropriately deal with the particularities and contingencies of patients’ individual cases and to protect the physician from inadmissible interventions (20). In doing so, the physician should not be guided by influences that are not based on the best medical reasoning. As the ICoME states in its sixth principle: “Physicians must take responsibility for their individual medical decisions and must not alter their sound professional medical judgments on the basis of instructions contrary to medical considerations” (19) This is the area of the physician’s individual decision-making autonomy that is of interest to AI support within clinical decision-making.

The goal of promoting the health and well-being of the unique patient with his or her individual circumstances, wishes and preferences cannot be completely regulated by general guidelines. Deviating from general rules and guidelines is therefore ethically permissible and even imperative because it allows for a more person- and context-specific treatment that is more in line with the patient’s well-being and autonomy. The decision-making autonomy of the physician should therefore serve to better fulfill the moral obligations of the medical ethos in the best interest of the patient.

This inherent moral orientation of medical practice should already make quite clear that the physician’s professional decision-making autonomy is by no means a matter of arbitrariness. In this respect, the physician’s professional decision-making autonomy is fundamentally different from the autonomy of a citizen or patient over his or her way of life. The patient’s decision-making autonomy is not to be respected in order to pursue a goal that can be determined in terms of content on a supra-individual basis, but in order to choose the preferred way of life and to shape it according to his or her own values, wishes and commitments. In this way, the individual is enabled to shape his or her life in a self-determined way and usually encounter protected limits only where he or she threats to compromise the freedom of others (20). Respecting a patient’s autonomy means that he or she may decide—even against rational reasons—to the point of arbitrariness. The professional autonomy of the physician, however, is something quite different. It enjoys its moral legitimacy only insofar as it is exercised to serve the goal of promoting the patient’s health and well-being (20). The physician is granted autonomy as a discretionary power because it serves to better fulfill this purpose, not for its own sake. It is a purposeful autonomy. A doctor does not have the right to arbitrariness and the right to recommend something other than what makes medical sense.

What follows from this conception of the physician’s decision-making autonomy is that it is not per se non-negotiable, but that its necessity could be replaced by, or the discretionary scope could be limited due to more appropriate means to pursue the moral obligations of medical practice. If its purpose is better achieved by other means, the physician’s decision-making autonomy may justifiably be limited. And it is precisely this potentially legitimate replacement or restriction of the physician’s decision-making autonomy through the introduction of any form of standardization or technology that is constantly under discussion. However, before we turn to the question of how AI support affects decision-making autonomy and whether it is a legitimate restriction, we must first explain what criteria make a restriction of the physician’s decision-making autonomy legitimate from an ethical perspective:

Since decision-making autonomy is granted to the medical profession only by reason of its main purpose of benefiting the well-being and will of the patient, no categorical argument against any limitation or interference by it follows. Such standardization, measures and technologies, which would increase the benefit for the patient, are therefore in line with the moral obligations of the medical profession and therefore constitute a weighty argument for a limitation of the physician’s decision-making autonomy. It could even be said that the use of them is imperative, as long as they serve the well-being and will of the patient in a better way. On the contrary, such standardization, measures and technologies that could knowingly harm the patient would be opposed to the moral obligations of the medical profession or at least conflict with them. The decisive criterion to which the physician’s decision-making autonomy must bow from an individual ethical point of view is therefore the best interest of the patient.

This can be supplemented by ethical reasons, such as the careful use of resources for reasons of a fair allocation of scarce resources. Such circumstances may constitute ethical reasons that may indeed allow individual physician decision-making autonomy to be restricted. However, these will not be pursued in more detail here, based on the assumption that medical practice must first and foremost be oriented toward the individual and his or her well-being and will (see ICoME) (19), and that questions of justice can only be negotiated to a very limited extent at this level.

In a nutshell, the ethical orientation of medical practice urges the physician to search for the best treatment corresponding the well-being and will of the patient. The physician’s decision-making autonomy is an expedient means to accomplish this purpose.

## 3 Discussing the impact of AI support on the decision-making autonomy of physicians

The use of AI support is essentially to be understood as an attempt to implement the compilation and extraction of knowledge appropriately to the structures of modern medicine in order to optimize the benefit for the patient. In view of the rapid speed of knowledge increase (and the equally rapid obsolescence of knowledge) as well as the increasing complexity of today’s healthcare, a physician as an individual is confronted with the challenge to derive the right rules of action from the immense medical knowledge. Accordingly, the first Hippocratic Aphorisms already postulated: ars longa, vita brevis—art is long, life is short. This disproportion between the length of a life and the extent of the medical art has become increasingly aggravated with modernity. Fewer and fewer parts of medicine can be learned and applied in a lifetime, even with increased life expectancy. The use of AI support could gradually reduce this tension.

Information technology systems that use AI, i.e., non-rule-based algorithms, recognize patterns and regularities (e.g., normal cases, typical progressions, and deviations) within the training data offered to the algorithm. The patterns and regularities identified in form of correlations enable the AI tool to compare the “learned” correlations with other data sets that were previously unknown to the AI tool and to make probability statements about the occurrence of a defined target in that unknown data set. Since the learned probability statements are sometimes empirically unidentified correlations or correlations that have not or hardly been taken into account in medical practice, such systems could—assuming that they have a sufficiently large database at their disposal—extrapolate individually tailored or “personalized” results with regard to the patient. The currently existing systems differ regarding the degree of their human “supervision” when obtaining the training data and learning weightings (c.f. supervised learning, unsupervised learning, and reinforcement learning).

The development and use are based at least on the assumptions that AI tools are competent to use significantly more complex data sets, thereby achieving better outcomes for the patient, and in a more efficient way than would be possible for human decision-makers, even more so in the short time available. And prima facie, under this assumption, there is little to be said against the use of AI support from an ethical point of view: if it can be proven for certain clinical decision-making situations that human decision-makers arrive at individually better decisions in terms of the patient’s well-being and will with the help of AI support than without its use, then the use of AI support is legitimized on the basis of this benefit—even if it means limiting the performance of physician’s decision-making autonomy. If the use of AI support serves the pursuit of the moral obligations of medical practice for an individual patient, its use is justified, and the physician does not have the right to ignore it. However, the physician has the right (and might sometimes have the moral obligation) not to follow the recommendations of an AI support if he or she has arguments that it does not recommend the best for the individual case.

Even though AI tools with these assumptions are only one more step in a whole series of measures to address the challenges of contemporary medicine (cf. institutional best practices, evidence-based guidelines, expert systems, etc.) and thus raise many already known problems, they could, according to Char et al., have “the potential to become the tipping point where a quantitative difference in autonomy becomes a qualitative problem” (21). Furthermore, AI support tools have also a disruptive potential for medical practice due to their inherent way of working, which distinguishes them from previous measures.

Thus, the question is whether and to what extent the use of AI support limits the physician’s decision-making autonomy and whether such a limitation is a more/less appropriate means to pursue the moral obligations of medical practice. However, as it is the case for patient’s decision-making autonomy, which goes beyond the mere freedom from external interventions and requires, for example, mental competences, adequate information, and understanding of this information as well as basic conditions that allow for a voluntary decision (22), some conditions are also required to enable the physician’s decision-making autonomy within the use of AI support (17). Frequently, both in the conceptual debate (23, 24) and in empirical interview studies (6, 8–10, 13), the question of physician’s autonomy is also answered with his or her possibility of taking responsibility for an intervention. The prerequisites include that the physician is capable to assess the patient’s situation and concerns and to recommend or provide appropriate treatment, that he or she is guided in his or her decisions by the best available medical evidence and professional standards, and that—if the patient is competent—he or she is able to engage in a process of shared decision-making. These conditions for the possibility to perform physician’s decision-making autonomy will now be discussed in more detail and the extent to which the use of AI tools threaten to compromise them will be shown.

### 3.1 Information: the physician’s capability to assess AI support and its action-guiding justification

Like any tool and measure, AI support systems have limitations that come with their way of working (see above) and can be a source of harm for patients.^3^

Already known are possible maldistributions and biases in the underlying training data from which AI tools “learn” their calculation paths and weightings to achieve the predefined goal. The training data serve as ground truth for the algorithm, as its information base about reality. If there are any biases in the training data, for example, due to a lack of representativeness of the data sets fed into the algorithm or simply due to the fact that only what can be measured or operationalized is depicted, this has consequences for non-represented persons or non-recorded parameters that may be important for persons: they simply do not exist for the AI.

Furthermore, AI tools perform inductive reasoning, especially based on large amounts of data collected in the past, and use correlations or patterns obtained from it to generate probability statements for new data sets. This way of working is particularly at risk for “false conclusions” from a human point of view. Correlations detected by the algorithm may be used for matching with new patient data sets, without necessarily being checked for plausibility or sometimes not even being able to be checked due to their complexity.

Limitations as such do not constitute a categorical objection against the use of a tool or measure; limitations of interventions are rather the rule in medical practice. However, the principles of beneficence and non-maleficence make it necessary to deal with them responsibly and appropriately in order to best benefit the patient regardless of their existence. For the use of AI support this means that there will probably never be “the perfect training data” and we always have to make compromises. Therefore, the following question arises from an ethical point of view: How good must the training data at least be in order to make medical decisions about a patient’s quality and length of life on this basis? Because training data will never be equally good for everyone, there is the additional in ethics well-known question: Is the acceptance of more harm for one group of people justifiable in view of the great benefit for a larger group of people, and if so, under what conditions? Since the two forms of limitations presented here as examples (biases and false conclusions) can result in harm to certain patients or patient groups, the fundamental orientation of medical practice to benefit the well-being and will^4^ of the individual patient means that these known limitations should be avoided as far as possible.

In order to assess whether the execution of an AI support recommendation is justified or even required in the case at hand, or whether a deviation from it would be justified or even required, the physician must be able to “handle” the AI statement in an action-oriented manner. To do this, he or she needs to assess how well the AI-generated recommendation meets the well-being of the individual patient. The physician will have to “merge” the recommendation of the AI with the necessary variabilities of the individual case, including those that may be more difficult to feed into the AI support tool or may not be fed into it at all. Necessary variabilities that must be taken into account are those that result from the characteristics, preferences and abilities of the patient in question. Other variabilities are not to be taken into account, if they are rather based on shortcomings in the medical decision-making process or on monetary incentives in the reimbursement system (20).

The physician must be epistemically enabled for both tasks, that is, the assessment of the adequacy of an AI statement as well as the supplementary consideration of other relevant variabilities of the individual case. In the first place, he or she needs a sufficient informational basis for the validation of AI support statement in individual case situations (cf. the debate on explicability, interpretability and transparency of AI tools for health care) (2, 25–28).

To prevent misunderstandings at this point: It is not necessarily a question of every recommendation and its origin being comprehensively “understood” from a medical point of view. From an ethical perspective, it is only imperative to serve the well-being of a patient in his or her individual situation as best as possible. For this ethical imperative, it is completely irrelevant for what medical reason this is achieved. At the latest since the progresses of evidence-based medicine (EbM), the proof of potential benefit has been measured by practical success through the processing of the best external evidence, preferably through meta-analyses of meaningful clinical studies. Since then, the focus has been in medicine on action-guiding knowledge, not explicative knowledge.

Yet, however, explicative knowledge can help the physician with the contextualization and possible validation of a recommendation for the concrete individual case. For example, explicative knowledge can help to exclude limitations that may harm certain people or groups to which the patient belongs. Defenders of EbM were also well aware of this at the time of its introduction: The physician’s central task according to the standard of EBM lies in the integration of medical (experience) knowledge about the individual case with the best available knowledge from clinical studies (29).

In the face of AI support, this is changing in that the form of data evaluation is taking place in a new way, one that is difficult for humans to comprehend, and it is moving alongside the methods already used to achieve action-guiding knowledge (e.g., guidelines from medical professional societies, medical-theoretical expertise). Particularly in the case of divergent or disagreeing action-guiding knowledge, decision-making situations can arise that are difficult to resolve from a human point of view without providing underlying reasoning (14, 30, 31), one of the central empirically identified barriers to the use of AI support [cf. (8, 9, 32)]. The moral orientation of medical practice to promote the patient’s well-being and prevent harm mandates that physician’s judgments should be sound wherever possible if harm can thereby be prevented. As pointed out by Amann et al. (2), it has already been argued in the discussion that medical AI based solely on validated performance is ethically defensible, even if the causal mechanisms behind a particular intervention prescribed by AI support remain opaque to the physician (25). According to Amann et al., however, this is no excuse for not providing explanations, which are an important prerequisite for sound clinical judgment, if such an explanation is indeed possible (2). Recent advances in elucidating the key features of AI models would establish a prima facie ethical obligation to reduce opacity and improve interpretability of AI support (2). Amann et al. concluded: “Failure to do so would mean intentionally undermining a physician’s capacity to control for possible misclassifications of individual clinical cases due, for instance, to excessive bias or variance in training datasets” (2).

What does it take to enable physicians to exercise their decision-making autonomy when dealing with AI statements? Firstly, before the clinician can meaningfully engage with the action-guiding statements provided by the AI support, there must be a reliable evaluation of the AI support tool itself, which systematically evaluates and oversees the underlying dataset, its data quality and the evidence provided on the positive proof of benefit of using the AI tool. Here, there are still new challenges for the review process, for example, by governmental or institutional review boards (21). Afterward, in order for the physician to be able to assess which form of action-guiding knowledge will best benefit the individual patient, sufficient informational conditions are required on the part of the AI support that allow the quality and validity of AI recommendations to be assessed. This necessary information varies in different fields of application, for example due to the consequences of a clinical decision on the patient’s life [cf. for this elsewhere (14)]. In such a way, a recommendation can be made for the individual patient that best corresponds to his or her well-being and avoids the inherent shortcomings of AI support tools.

### 3.2 Competence: the physician’s capability to integrate AI support into shared decision-making

A sufficient information base about the AI support alone is not sufficient for the exercise of physician’s decision-making autonomy as such information has to be properly integrated into the physician’s reasoning in clinical decision-making settings. The physician therefore needs additional competencies in dealing with different kinds of action-guiding knowledge, of which AI support is one.

Using AI in the medical decision-making process has an impact on informed consent and shared decision-making. This is because the information to be communicated must include the reasons underlying the physician’s recommendations of a therapy. However, in the case of AI, such reasons are sometimes very complex or inaccessible due to its opacity. But again, no arguments are visible as to why ethical norms should change with respect to informed consent. Rather, it is also true here that informed consent requirements must be implemented when using AI. This necessitates increased communicative requirements and needs in order to make the complexity of the decision-making steps to be informed and their (technical) occurrence comprehensible to patients in such a way that they are enabled to give informed consent.

Statements of AI support tools could guide physicians’ decisions “more than they are aware of since their outputs affect, shape, and even stand in tension with [the physician’s] judgments, thus raising questions on who is truly guiding the decision-making process” (33). This is especially the case for divergent or contradictory judgments where the recommendations are each based on different sources of action-guiding knowledge (e.g., AI support, guidelines from medical societies, medical theory). For example, if the relevant medical evidence-based guideline recommends a certain treatment as a priority that does not coincide with the recommendation of the AI supports, the physician may find herself or himself in a position where it is difficult to resolve the disagreement underlying these recommendations. This challenge is mainly discussed in the literature under the term “peer disagreement” (14, 30, 31). If the physician does not find himself or herself capable of resolving such a disagreement, he or she may see only the option of disclosing the divergent recommendations to the patient so that he or she can select his or her preferred form of knowledge reasoning.

What does this have to do with the physician’s decision-making autonomy? As already elaborated, the physician’s decision-making autonomy serves the purpose of best serving the patient’s well-being and will. For this purpose, the physician has not only to judge which of the action-guiding knowledge in the patient’s individual case is most likely to serve his or her well-being, but his or her judgment must also be brought together with the patient’s preferences within shared decision-making. Otherwise, there is a threat of paternalism through impersonal implementation of AI-generated recommendations against the preferences of the patient (34, 35). The physician is confronted with the communicative-practical challenge to mediate the patient’s will with the different options for action, which in turn are based on different forms of action-guiding knowledge. If the physician does not know the rationale for AI support recommendations or if the rationale remains only a statement about the evidence-based performance of the AI support tool, a recommendation contrary to the physician’s judgment may, in very practical terms, limit the physician’s discretion to suggest those interventions that, in the physician’s judgment, best serve the patient’s interests and will. As already known from fields other than healthcare, a person who disagrees with an AI recommendation often needs to present far more and higher quality evidence to disprove the AI statement than the evidence used to create that statement (21). It is easier to agree with a recommendation from AI than to disagree. Physicians interviewed also see a real threat that the accuracy of their judgment and decision-making skills could be questioned in the face of AI statements (7, 10). Such hurdles to resolving contradictions can discourage people from challenging algorithmic outputs (21, 36). It becomes problematic when the normative orientation towards the well-being and will of the patient is no longer the decisive factor, but rather such psychological inadequacies.

Thus, in view of the handling of AI support, being competent to operate in clinical decision-making setting demands more from physicians than becoming an “information specialist” (24). A selection of useful competencies that are also relevant to the exercise of physician’s decision-making autonomy has been compiled by Sand et al. (24): “1. Reporting and informing about sensitivity rates and experimental performance; 2. Understanding reasonable output; 3. Understanding input data (e.g., relationship between image quality and accuracy rate); 4. Awareness of impact of utilizing medical AIs on one’s own skills, and capacities; 5. Awareness of task specificity of the medical AI; 6. Assessing, monitoring and reporting of outputs over time.” In addition to medical skills, such competencies could help to enable physicians to assess the potential and limits of their own judgment and that of AI supports more realistically and to put them into a well-reasoned relationship. In addition, however, extensive communicative skills are required to explain this to the patient for his or her informed consent and to adequately address the aspects relevant to the patient’s decision.

If either of these two conditions, namely the provision of information by the AI support tool (cf. section 3.1) or the competencies of the physician (cf. section 3.2), is missing, this can compromise the performance of physician’s decision-making autonomy, insofar as it deprives the physician of the opportunity to meaningfully assess the appropriateness of an AI statement in terms of its suitability to the well-being and will of the individual patient with his or her specific circumstances. If the physician is not in a position to do so, he or she cannot validate or control the justification of an AI statement.

### 3.3 Voluntariness: the structural and institutional conditions of the use of AI support

The use of medical AI tools takes place within an institutionalized healthcare system. This context, which particularly concerns the working and financing conditions of medical practice, is crucial for enabling the first two conditions even though it is not exclusive or new for the use of AI.

Particularly, these conditions refer to the institutionally guaranteed and unscathed possibility of the physician of being able to choose between alternative courses of action on the basis of his or her own medical judgment about the most appropriate measures to fulfill the goals of medical practice. According to the fifth paragraph of the ICoME, physicians must wherever possible “not allow their individual professional judgment to be influenced by the possibility of benefit to themselves or their institution” (19). This also includes not to be a subject to institutional constraints that mandate the execution of the AI-generated recommendation, for example, for non-medical reasons (8). The execution of an AI-generated recommendation should only be imperative if it meets the purpose of medical practice better than alternative actions. Although, in our best knowledge, no one in the discussion is currently calling for physicians to be obliged to follow recommendations made by an AI support tool (instead, physicians’ judgment is being called for to review AI recommendations). Nevertheless, consideration should be given to the question of how physician’s judgments should be dealt with if they arrive at divergent assessments despite taking AI recommendations into account (cf. section 3.2). Unless solid legal safeguards are put in place for physicians, the use of AI support may become quasi-commands for fear of justiciable consequences of deviations from the AI support.

However, more subtle forms that may resemble practical constraints under the given circumstances should not remain unconsidered. For example, given a shortage of staff and time, the standard use of AI tools at the same time may leave insufficient space for appropriate physician review and judgment about compliance or non-compliance with the AI recommendation. Also, certain monetary incentives could make the additional review of AI recommendations more difficult and therefore require a high level of personal resistance on the part of physicians to evade such general conditions. The implementation should also be accompanied by a critical approach to the technical possibility of using AI tools in pursuit of optimizing workforce performance indicators and to recommend decisions in favor of financial yield maximization (21). Although, these interests are not compatible with the main ethical principles of medical practice, such rationalizations already determine it in many ways. While the goals pursued by the physician may well correspond to the normatively required goals of medical practice in that they aim to improve the patient benefit through the use of AI tools, they could possibly differ from the goals of the purchasers or institutions (21).

One of the central hopes associated with the use of AI support—and almost every recent introduction of digital technologies—is therefore increased efficiency and the resulting time savings for other activities (6, 11). The time saved could then, it is hoped, be used as a positive contribution to patient benefit to maintain relationships and more communicate with patients and to “make healthcare human again” (37). Such a horizon of a successful implementation of AI support can serve as a foil to recognize and criticize current grievances. The aim and standard of medical practice—also in digitalized settings—remains the patient’s well-being and will. However, in order to prevent physicians from increased work load through the use of AI support and the adoption of more profitable activities, flanking efforts are needed at the institutional and policy levels (37).

## 4 Conclusion

The physician’s decision-making autonomy is not a freedom to treat patients as he or she wishes, but is fundamentally rooted in the medical ethos to promote the patient’s health and well-being, thereby respecting the patient’s autonomy and rights. The role of a physician in ethical terms does not change due to AI. It is a tightrope to tread in order to meet the moral obligations toward the respective patient in the best possible way. The moral principles of medicine also apply unconditionally to the use of AI. The use of AI support must be assessed and weighed on the basis of these moral principles. AI support has the potential to better inform decision-making and thus indirectly promote the physician’s decision-making autonomy, and its use should be pursued where it succeeds. The functioning and information processing of AI support should be designed in a way that supports the decision-making autonomy of the physician; however, it should not be designed and implemented in such a way that, although it assumes the need for physician autonomy (e.g., in the form of a required medical review of the AI statement), the use of AI support actually exceeds the capabilities of the physician. Physicians also have a crucial role to play here: they should neither uncritically accept nor inappropriately resist the potentials of AI for healthcare, but actively engage in the discourse and development and critically examine whether the integration of AI tools in concrete fields of application has the potential to improve or impede the main goals of medical practice. Nonetheless, many stakeholders involved in design and development have a crucial role to play here, as is initially pursued in approaches such as user-centered design, HCD, participatory design, co-design, and value-sensitive design, all of which place more emphasis on the context-sensitivity of decision-making processes [c.f. e.g., (38)]. The implementation and use of AI support, like any new standardization, measure and technology, will ultimately be judged from an ethical point of view against the main function of the physician’s decision-making autonomy: the best interest of the patient. If the physician should fulfill his or her moral obligation to promote the health and well-being of the patient, then the use of AI should be designed and implemented in such a way that it promotes or at least maintains the physician’s decision-making autonomy.

## Data availability statement

The original contributions presented in the study are included in the article/supplementary material; further inquiries can be directed to the corresponding author.

## Author contributions

FF: Writing – original draft, Writing – review & editing. UW: Writing – review & editing.

## Footnotes

^1^In this respect, the debate about possible “replacements” of doctors by AI is not of interest here, even if such could limit the autonomy of the medical profession. For the (medical) ethical discussion, it is irrelevant whether professions or workplaces are reduced or changed, as long as moral obligations toward the patients do not (potentially) suffer as a result.

^2^This fundamental orientation of medical practice was also reaffirmed in the revised version of the ICoME of 2022. According to it, the primary duty of the physician is “to promote the health and well-being of individual patients by providing competent, timely and compassionate care consistent with good medical practice and professionalism.” And the physician “must provide care with the utmost respect for human life and dignity, and for the autonomy and rights of the patient” (19).

^3^The limitations are presented here in a simplified form and do not necessarily apply equally to every AI tool. However, they serve to illustrate that known limitations can exist in this or a similar way when using AI tools. Although there are fortunately attempts at technical solutions to reduce the limitations described here, AI tools cannot, as far as we know, be fundamentally relieved of them.

^4^From here on, for reasons of better comprehensibility, the will of the patient or his treatment preferences will be excluded for the moment (*cf.* then again 3.2). As mentioned at the beginning, every medical recommendation by the physician presupposes that it is subsequently subjected to the process of shared decision-making.
